# Bioinformatics analysis and experimental verification of the cancer-promoting effect of DHODH in clear cell renal cell carcinoma

**DOI:** 10.1038/s41598-024-62738-0

**Published:** 2024-05-25

**Authors:** Songsong Wang, Yan Li, Yilong Lin, Junting Li, Lang Guo, Haoyu Wang, Xinyuan Lin, Ziming Liu, Bingqi Zhang, Zhengming Liao, Zhongmin Zhang

**Affiliations:** 1 Department of Urology, Zhongshan Hospital of Xiamen University, School of Medicine, Xiamen University, Xiamen, 361000 China; 2 School of Medicine, Xiamen University, Xiamen, 361000 China; 3 Department of Urology, Hubei Provincial Hospital of Traditional Chinese Medicine, Wuhan, 430061 Hubei China; 4 Hubei Key Laboratory of Theory and Application Research of Liver and Kidney in Traditional Chinese Medicine (Hubei Province Hospital of Traditional Chinese Medicine), Wuhan, 430061 China; 5 The First Clinical Medical Institute, Hubei University of Chinese Medicine, Wuhan, 430060 China; 6 The First Affiliated Hospital of Xiamen University, School of Medicine, Xiamen University, Xiamen, 361000 China; 7 College of Integrative Medicine, Fujian University of Traditional Chinese Medicine, Fuzhou, 350000 China

**Keywords:** Apoptosis, Bioinformatics, Clear cell renal cell carcinoma, DHODH, Ferroptosis, Immunotherapy, Cancer, Cell biology, Computational biology and bioinformatics, Molecular biology, Diseases, Nephrology, Oncology, Pathogenesis, Urology

## Abstract

Clear cell renal cell carcinoma (ccRCC) is a malignant tumor of the urinary system. To explore the potential mechanisms of DHODH in ccRCC, we analyzed its molecular characteristics using public databases. TCGA pan-cancer dataset was used to analyze DHODH expression in different cancer types and TCGA ccRCC dataset was used to assess differential expression, prognosis correlation, immune infiltration, single-gene, and functional enrichment due to DHODH. The GSCALite and CellMiner databases were employed to explore drugs and perform molecular docking analysis with DHODH. Protein–protein interaction networks and ceRNA regulatory networks of DHODH were constructed using multiple databases. The effect of DHODH on ccRCC was confirmed in vitro. DHODH was highly expressed in ccRCC. Immune infiltration analysis revealed that DHODH may be involved in regulating the infiltration of immunosuppressive cells such as Tregs. Notably, DHODH influenced ccRCC progression by forming regulatory networks with molecules, such as hsa-miR-26b-5p and UMPS and significantly enhanced the malignant characteristics of ccRCC cells. Several drugs, such as lapatinib, silmitasertib, itraconazole, and dasatinib, were sensitive to DHODH expression and exhibited strong molecular binding with it. Thus, DHODH may promote ccRCC progression and is a candidate effective therapeutic target for ccRCC.

## Introduction

Renal cell carcinoma (RCC) is one of the top 10 most common cancers worldwide^1^. Clear cell RCC (ccRCC) is a malignant tumor of the urinary system that accounts for more than 80% of all RCC cases. Thus, ccRCC is the most common histological subtype among malignant kidney tumors^2^. The incidence rate of ccRCC has been increasing annually, with 30% of patients experiencing distant metastasis upon diagnosis and 60% dying within 1–2 years of diagnosis^3^. Although surgery is the primary treatment option, immune checkpoint blockade therapy using immunotherapeutic agents is the first-line clinical treatment for most patients with advanced-stage tumors^4^. Although the development of immune checkpoint inhibitors has been rapid, that of biomarkers has been relatively slow. The absence of consistent biomarkers hampers the selection of immune checkpoint inhibitors^5^. PDL1 remains the most used immune biomarker for various solid tumors; however, its predictive role in the immunotherapeutic response of patients with metastatic RCC remains to be validated^6^. Furthermore, the effect of targeted therapies and immune modulators on the quality of life of patients with RCC remains unclear^7^. Thus, in addition to reliable therapeutic targets, prognostic and predictive biomarkers are currently lacking for patients with RCC^8^.

Dihydroorotate dehydrogenase (DHODH) is an iron-containing flavoenzyme located in the mitochondrial inner membrane and plays a crucial role in de novo pyrimidine synthesis^9^. Gene encoding DHODH is also a ferroptosis-associated gene that synergizes with mitochondrial GPX4 to inhibit ferroptosis by reducing ubiquinone (CoQ) to ubiquinol (CoQH2). DHODH inhibitors can block tumor growth by inducing ferroptosis^10^. Notably, DHODH is an independent risk factor for neuroblastoma, and high DHODH expression is significantly associated with a poor prognosis^11^. The binding of DHODH with β-catenin promotes esophageal squamous cell carcinoma cell proliferation^12^ and drives nucleotide synthesis to promote liver metastasis of colorectal cancer^13^. Multiple studies have shown that DHODH inhibitors can kill tumor cells and significantly inhibit the growth of various tumor types^14^. DHODH inhibitors can block the cell cycle of glioblastoma^15^, breast cancer^16^, and myelodysplastic syndrome cells^17^, and inhibit their proliferation. Furthermore, DHODH can increase the sensitivity of liver cancer cells to chemotherapeutic drugs^18^. Accordingly, DHODH is considered an important therapeutic target for cancer^19^. The potential regulatory role of DHODH in the cell cycle, ferroptosis, and resistance of different tumor cells is also crucial. However, studies on the effects of DHODH on ccRCC are lacking.

To fill this gap in existing literature, in this study, we used various public databases and analytical methods to examine differential expression of DHODH in ccRCC and normal tissues. We explored the potential function of DHODH and its effect on immune cell infiltration in ccRCC. In addition, we performed drug target analyses to identify candidate compounds that could target DHODH for ccRCC treatment. In vitro experiments were conducted to confirm the regulatory function of DHODH in ccRCC cell proliferation, migration, apoptosis, and ferroptosis. In addition to highlighting the role of DHODH in ccRCC development, this study demonstrates its potential as a therapeutic target.

## Methods

### DHODH gene information

We used the GeneCards database^20^ (https://www.genecards.org/) to visualize the chromosome and subcellular locations of gene encoding DHODH. The protein topology of DHODH was obtained from PROTTER^21^ (https://wlab.ethz.ch/protter/start/). The RNA expression levels of the DHODH gene in 20 normal tissues were obtained from the NCBI database (https://www.ncbi.nlm.nih.gov/).

### Data collection and preprocessing

RNA-seq data processed using the STAR pipeline from 33 tumor projects were downloaded and extracted in TPM format from TCGA database (https://portal.gdc.cancer.gov). RNA-seq data processed using the Toil pipeline^22^ from TCGA, and GTEx in TPM format were obtained from UCSC XENA (https://xenabrowser.net/datapages/). The corresponding TCGA data for pan-cancer and GTEx data for normal tissues were collected. The ccRCC datasets, GSE12606^23^ and GSE189331^24^, were obtained from the GEO database (https://www.ncbi.nlm.nih.gov/geo/).

To validate the expression of DHODH in pan-cancer, the TIMER database^25^ (http://timer.cistrome.org/) was used to visualize the differential expression of the DHODH gene across various cancer types. The effect of DHODH on pan-cancer outcomes was assessed using the TIMER database.

### DHODH expression

Using the Stats and Car packages in R (version 4.2.1), Wilcoxon rank-sum test analysis was conducted to examine the differential mRNA expression of DHODH across all cancers. Differences in the mRNA expression levels of DHODH were also assessed in different disease states (tumor or normal) and cancer stages (including TNM staging and histological grading) of ccRCC, and visualized using box plots, violin plots, and paired sample line plots with the ggplot2 package. The data were standardized using the normalizeBetweenArrays function in the Limma package. Differentially expressed genes (DEGs) were selected based on the criteria: "|Log2(Fold Change)|≥ 1 and *P* value < 0.05." Volcano plots were used to display the expression of DHODH in the GSE12606 dataset. The specific antibody against DHODH, HPA-010123, was obtained from the Human Protein Atlas (HPA, http://www.Proteinatlas.org/) online database^26^ for immunohistochemical staining of clinical ccRCC and normal kidney tissues.

### Kaplan–Meier plot analysis

By employing the survival package, Kaplan–Meier survival analysis and log-rank tests were performed using TCGA-KIRC dataset to investigate the relationship between gene expression and overall survival (OS), progression-free interval (PFI), and disease-specific survival (DSS) in patients with ccRCC. Subgroup survival analyses based on TNM stage and histological grade were also conducted, and the results were visualized using the Survminer and ggplot2 packages.

### Immune cell infiltration analysis

Using the single-sample Gene Set Enrichment Analysis (ssGSEA) algorithm from the GSVA package (version 3.6)^27^, immune infiltration analysis was conducted using TCGA-KIRC dataset to assess the enrichment status of 24 immune cell types^28^ across samples grouped by the expression levels of DHODH. The Wilcoxon rank-sum test was employed for this analysis. Thereafter, Spearman correlation analysis was performed to determine the effect of DHODH expression on immune cell infiltration, yielding Spearman correlation coefficients. The results were visualized using the Laplace method and grouped into violin plots. A focused examination was performed on the Spearman correlations between DHODH expression and the infiltration levels of six pivotal immune cell types (B cells, CD8 + T cells, macrophages, neutrophils, NK cells, and dendritic cells) using the circlize package; the results were displayed in chord diagrams.

The TISCH database was used to explore the single-cell expression of DHODH, and the TIMER 2.0 database was employed to determine the effect of DHODH expression on tumor immune cell infiltration in 115 different subtypes of tumors. The results are displayed in a circular clustering diagram.

The effect of the expression level of DHODH on the anticancer immune status in ccRCC was analyzed using the Tracking Tumor Immunophenotype (TIP) database^29^ (http://biocc.hrbmu.edu.cn/TIP). The immune activity scores between samples with high and low DHODH expression were compared using Student's *t*-test or Wilcoxon rank-sum test, and the results were visualized using the R packages, pheatmap and ggplot2. To comprehensively illustrate the anticancer immune status of samples with high and low DHODH expression, all samples were divided into high- and low-expression groups based on the median expression value of DHODH, and samples with expression values close to the mean value of each group were selected as examples (low-expression sample: TCGA-CW-6090-01A-11R-1672-07; high expression sample: TCGA-CZ-5453-01A-01R-1503-07). Thereafter, the effect of DHODH on the seven steps of antitumor immune response was analyzed. The correlation between DHODH expression and the immune checkpoint were analyzed using scatter plots^30^.

### Drug sensitivity analysis and molecular docking

To analyze small-molecule drugs targeting DHODH, the GSCALite online database^31^ was used to calculate the Spearman correlation coefficient to determine the correlation between DHODH expression levels and the drug sensitivity (50% inhibitory concentration (IC50)) of 65 small-molecule drugs from the GDSC and CTRP databases.

RNA expression data (RNA: RNA-seq) and drug data (compound activity: DTP NCI-60) of the NCI-60 cell lines were downloaded from the CellMiner database. The correlation between DHODH and small-molecule drugs was reanalyzed and presented as a dot plot. The sensitivity of small-molecule drugs in the DHODH high- and low-expression groups is displayed using a box plot.

For molecular docking, the name, molecular weight, and 3D structure of the small-molecule drugs were determined using the PubChem database, and the 3D structure corresponding to the DHODH gene was downloaded from the RCSB PDB database (https://www.rcsb.org/). Ligands and proteins were prepared for molecular docking using the AutoDock Vina software (http://vina.scripps.edu/). Finally, the AutoDock 1.5.6 software was used to dock the structure of DHODH with that of the small-molecule drug, where the affinity (kcal/mol) value represented their binding ability. The lower the affinity (kcal/mol), the more stable the binding between the ligand and receptor. The results were visualized using the PyMOL software.

### Single-gene differential expression and enrichment analysis

The statistical ranking of genes with DHODH expression higher or lower than the median was defined as the high or low DHODH expression groups, respectively. The DEGs between these two groups were identified using the DESeq2 R package^32^ and an unpaired Student's *t*-test. Genes with adjusted *P* value < 0.05 and |Log_2_(Fold Change)|≥ 1 were considered statistically significant and were included in the subsequent analysis. All DEGs are presented in volcano plots and heat maps.

To determine the function of DHODH, the DEGs were subjected to Gene Ontology (GO)^33^ and Kyoto Encyclopedia of Genes and Genomes (KEGG)^34^ analyses using the ClusterProfiler package^35^ in the R software. Statistical significance was set at *P* < 0.05. GO terms were divided into three categories: biological processes (BP), cellular components (CC), and molecular functions (MF). Some results were visualized using bar plots generated using the ggplot2 package.

Gene set enrichment analysis (GSEA)^36^ was performed for all DEGs with statistical significance. This analysis aimed to identify differences in functional phenotypes and signaling pathways between the high- and low-expression groups. The ClusterProfiler package was used for this analysis, and the reference gene set was obtained from the c2.cp.all.v2022.1. Hs.symbols.gmt gene set database within MSigDB Collections. *P* < 0.05 and normalized enrichment score (|NES|) > 1 indicated significant enrichment.

### Identification of the ceRNA and protein interaction (PPI) networks

To explore the potential molecular mechanisms of DHODH in ccRCC, StarBase (https://starbase.sysu.edu.cn/) and mirRTarBase (https://mirTarBase.cuhk.edu.cn/) were used to predict the miRNAs that can interact with DHODH and lncRNAs that can bind to miRNAs, respectively. To better understand the role of DHODH in ccRCC resistance, we compared the expression levels of miRNAs between sunitinib-resistant samples and sunitinib-sensitive samples in the GEO dataset, GSE189331, and selected differentially expressed miRNAs that were negatively correlated with DHODH expression (logFC <  − 1 and *P* < 0.05). Furthermore, based on TCGA-KIRC, Kaplan–Meier analysis was performed to identify prognostic lncRNAs in ccRCC and Cytoscape (version 3.10.0) was used to display the interactions among mRNAs, miRNAs, and lncRNAs to analyze the potential ceRNA regulatory mechanisms involved in ccRCC.

The STRING database (http://string-db.org) (version 12.0)^37^ was used to identify proteins that interact with DHODH. Thereafter, a PPI network with complex regulatory relationships was constructed. Interactions with medium confidence > 0.4 were considered statistically significant. The MCODE plugin in Cytoscape was used to analyze key functional modules. The selection criteria were as follows: K-core = 2, degree cutoff = 2, maximum depth = 100, and node-score cutoff = 0.2. Subsequently, KEGG and GO analyses were performed using these shortlisted genes.

### Cell culture and RNA interference

The human RCC cell lines, 786-O and OS-RC-2, were obtained from Procell Life Science&Technology Co., Ltd. (Procell, Wuhan, China) and cultured in Procell's basic medium (ROMI-1640, Procell, Wuhan, China) supplemented with 1% penicillin/streptomycin (MA0110, Meilunbio, Shanghai, China) and 10% fetal bovine serum (TIANHANG, Zhejiang, China). The 786-O and OS-RC-2 cells were maintained at 37 °C in a 5% CO_2_ environment.

DHODH-targeting siRNAs (hDHODH-259, hDHODH-849) were transfected into 786-O and OS-RC-2 cells for 48 h using the DharmaFECT^TM^1 transfection reagent kit (4000-3, Engreen Biosystem Co. Ltd.), according to the manufacturer’s instructions. The siRNAs targeting DHODH and scrambled siRNAs were obtained from Sangon Biotech (Wuhan, China).

### Cell proliferation and migration assays

First, 786-O and OS-RC-2 cells (10 × 10^4^ cells/well) were seeded in 6-well plates and cultured for 48 h. RNA interference (RNAi) technology was used to interfere with the expression of DHODH in 786-O and OS-RC-2 cells. On days 3 and 5 of cell culture, the 786-O and OS-RC-2 cells were digested with trypsin. The proliferation of 786-O and OS-RC-2 cells was detected using the Neubauer counting chamber cell counting method.

The EDU proliferation assay was performed using the BeyoClick™ EDU-488 Cell Proliferation Assay Kit (C0071S, Beyotime, Shanghai, CHN). Briefly, cells were seeded (3 × 10^4^ cells/well) in a 6-well plate containing the corresponding concentration of EDU reagent (1:500) for 3 h. The cells were then washed twice with 1 × PBS (BL302A; Biosharp, Anhui, CHN) for 5 min, incubated with 4% paraformaldehyde for 30 min, permeabilized with 0.3% Triton X-100 immunostaining permeabilization solution (BL935A; Biosharp, Anhui, CHN), and staining using the reaction solution. Images were captured using a fluorescence microscope.

The effect of DHODH on the colony forming ability of 786-O and OS-RC-2 cells was evaluated. 786-O and OS-RC-2 cells (500 cells per well) were seeded in 6-well plates, cultured for 1 week, and subjected to RNAi-mediated interference of DHODH expression. Cells were cultured for another week. The resulting colonies were fixed with 4% paraformaldehyde and stained with crystal violet solution.

For the wound healing assay, healthy 786-O and OS-RC-2 cells were seeded in 6-well plates (50 × 10^4^ cells/well). After 48 h culture, the RNAi technology was used to interfere with the expression of DHODH in 786-O and OS-RC-2 cells. A scratch was mechanically created using a 1 ml sterile pipette tip, and the cells were cultured for 24 and 48 h. Images were captured at 0, 24, and 48 h, and the wound healing rate was calculated using the ImageJ software.

For the transwell assay, cells were seeded (20 × 10^4^ cells/well) and cultured for 48 h. RNAi technology was used to interfere with the expression of DHODH in 786-O and OS-RC-2 cells. The 786-O and OS-RC-2 cells were digested with trypsin, and a cell suspension containing 2 × 10^4^ cells in 300 $\mu$L of complete culture medium containing 2% fetal bovine serum was seeded in the upper chamber. The lower chamber was filled with complete culture medium containing 10% fetal bovine serum. After 24 h culture, the cells were fixed with 4% paraformaldehyde, stained with crystal violet, and observed and quantified using ImageJ software to measure their invasion ability.

### Clinical tissue samples

Tumor tissues and the corresponding adjacent normal tissues were obtained from five patients with primary ccRCC who were admitted to the Urology Department of Zhongshan Hospital of Xiamen University in 2023. All patients with CRC underwent curative surgery, and their pathological diagnosis was ccRCC without other malignant tumors. None of the patients underwent preoperative radiotherapy, neoadjuvant chemotherapy, or other treatments. All patients signed a written informed consent form. The study was approved by the Medical Ethics Committee of Zhongshan Hospital of Xiamen University (2024-553). The study was conducted in accordance with the principles of the Declaration of Helsinki. All methods were performed in accordance with the relevant guidelines and regulations.

### Western blot analysis

Total protein was extracted from cells or tumor tissues using RIPA lysis buffer (BL504A; Biosharp, Anhui, CHN) supplemented with protease inhibitors and EDTA (Beyotime, Shanghai, CHN). Protein amounts were quantified using a BCA Protein Assay Kit (P0010; Beyotime, Shanghai, CHN). Equal amounts of protein were separated by performing SDS-PAGE (Beyotime, Shanghai, CHN) using on 10–12% resolving gels and were transferred onto a 0.2 μM PVDF membrane (Immobilon®-PSQ, Ireland). The membranes were blocked with 5% skim milk and incubated with the following primary antibodies: DHODH (14877-1-AP, Proteintech), N-cad (66219-1-lg, Proteintech), Vimentin (E-AB-18212, Proteintech), Snail (26183-1-AP, Proteintech), MMP2 (E-AB-40409, Proteintech), Parp1 (13371-1-AP, Proteintech), GPX4 (67763-1-lg, Proteintech), SLC7A11 (26864-1-AP, Proteintech), FTH1 (10727-1-AP, Proteintech), and GAPDH (60004-1-lg, Proteintech), diluted 1:1000 in Western primary antibody dilution solution (E-IR-R125, Elabscience, Wuhan, CHN). Thereafter, the membranes were incubated with the corresponding secondary antibodies, rabbit anti-mouse (SA00001-2-lg; Proteintech) and goat anti-rabbit (SA00001-2-lg; Proteintech), diluted 1:3000. The signal was detected using TanonTM Femto-sig ECL Western Blotting Substrate (180-506, Tanon).

### Expression of ferroptosis-related protein in renal tissue based on immunohistochemistry and transmission electron microscopy (TEM)

Conventional ccRCC tissues and normal renal tissues adjacent to the carcinoma were embedded in paraffin and sliced. The sliced tissues were incubated with 3% hydrogen peroxide at room temperature for 15 min and washed 3 times with PBS (3 min/wash). Following removal of the excess water, the tissues were incubated with the diluted primary antibodies, including those against FTH1, GPX4, and SLC7A11 (1:300, the product catalog number is the same as that for the western blotting experiment) overnight at 4 °C. Immunoreactive bands were stained according to the instructions of the immunohistochemical kit (Beijing Zhongshan Jinqiao Biotechnology Co., Ltd., PV-9000) and concentrated DAB kit (Beijing Suolaibao Technology Co., Ltd., DA1010).

After pre-treatment, cells were collected, precipitated, and fixed with 2.5% glutaraldehyde for 2 h at 4 °C. Images were captured after tissue dehydration, infiltration, slicing, embedding, and staining.

### Data analysis

The experimental data were analyzed using GraphPad Prism 9.3.0 software. The results are presented as mean ± standard deviation (mean ± SD). One-way analysis of variance (ANOVA) was used for multiple comparisons between groups. A *P* < 0.05 indicated statistical significance.

## Results

### DHODH localization, variants, and expression

**Figure 1:**
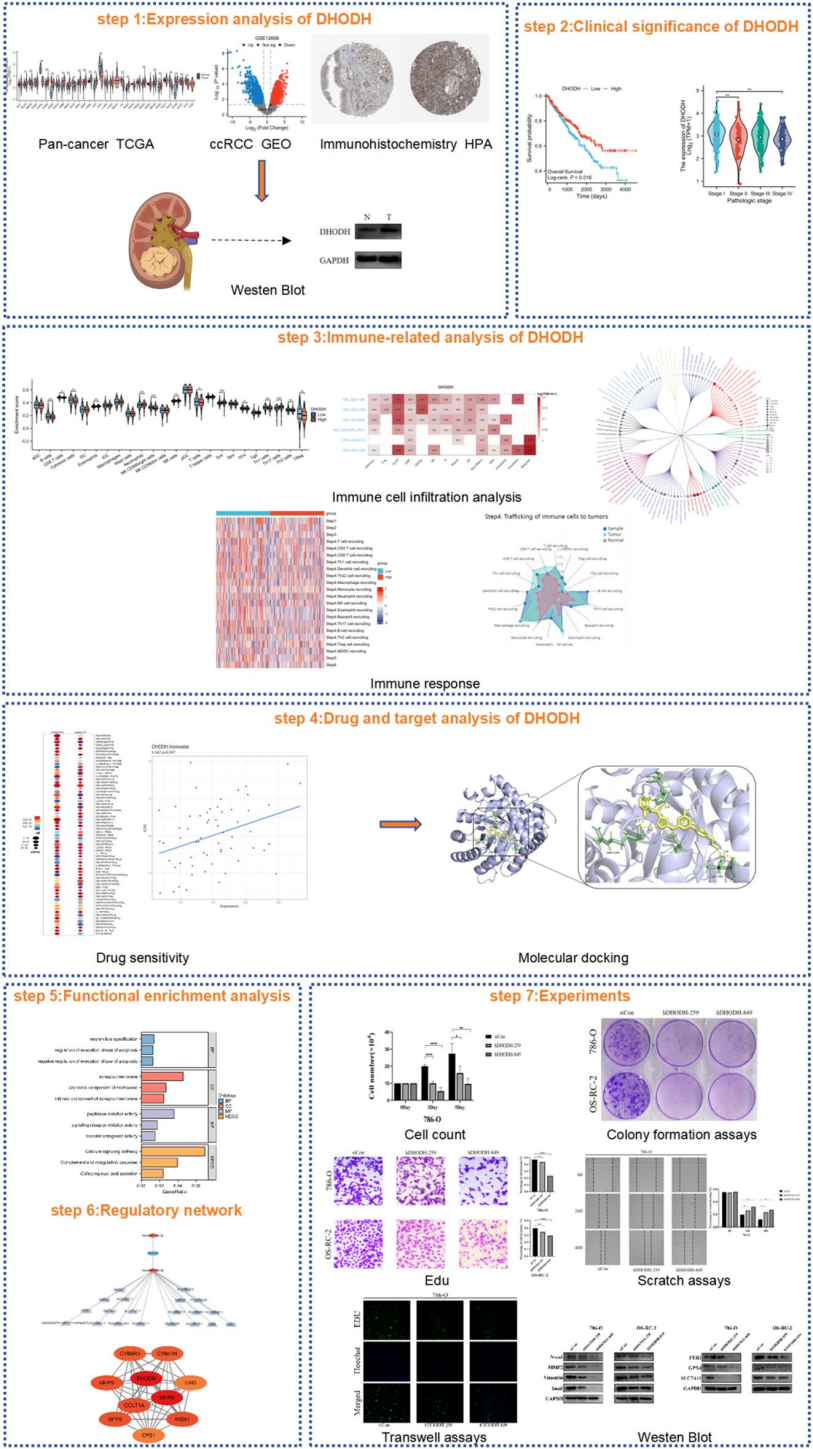
Overview of research design, to provide a more understandable research ideas.

**Figure 2:**
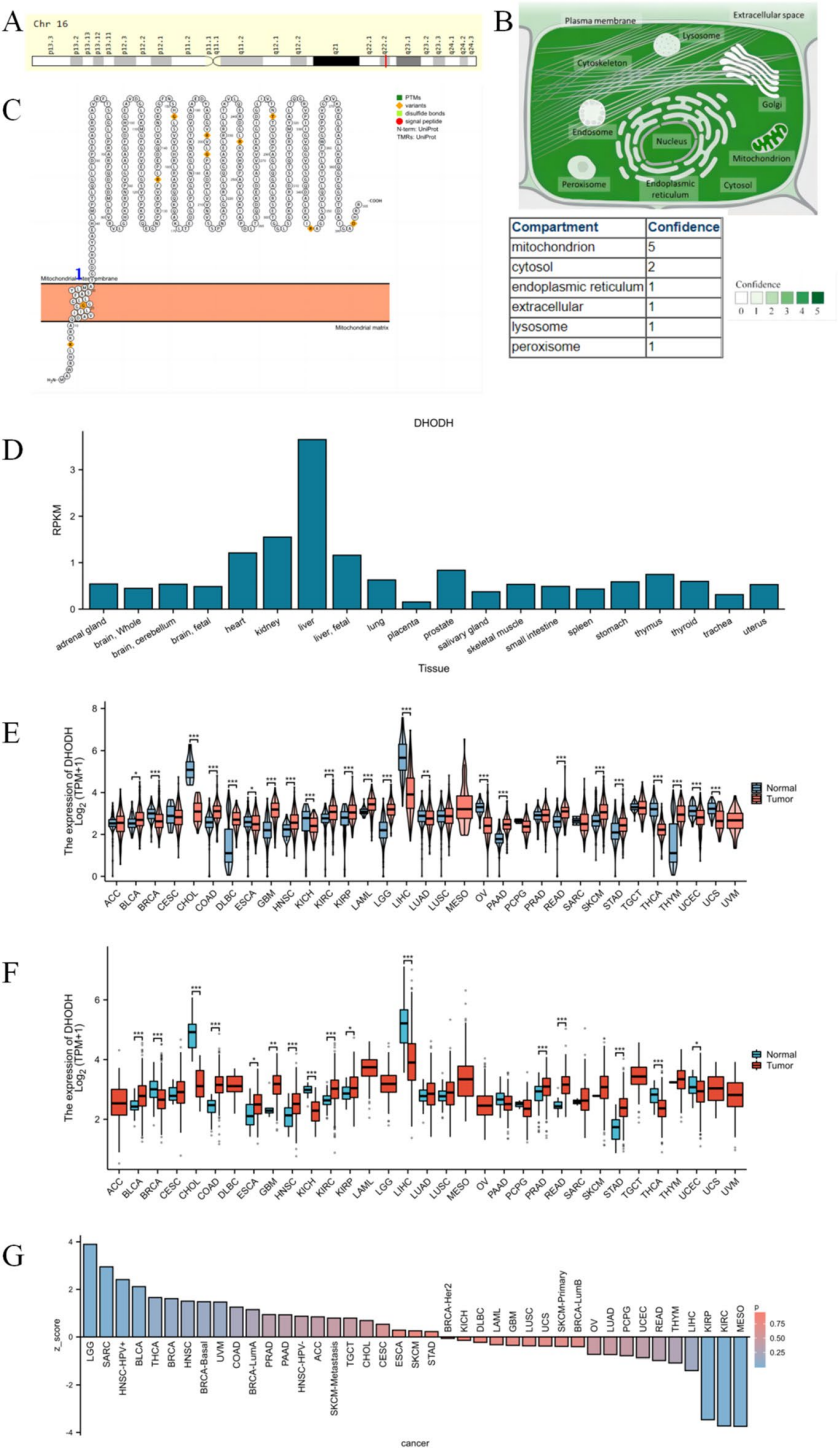
DHODH gene features and their pan-cancer relationship. (**A**) Chromosomal location of the DHODH gene; (**B**) Subcellular localization of the protein encoded by the DHODH gene, dihydroorotate dehydrogenase; (**C**) Topological structure of dihydroorotate dehydrogenase; (**D**) Expression of DHODH in various normal organs; (**E**) Pan-cancer differential expression of DHODH using TCGA database combined with the GTEx database; (**F**) Pan-cancer differential expression of DHODH using TCGA database; (**G**) Pan-cancer prognostic correlation of DHODH according to the TIMER database.

Overview of research design in Fig. 1. Gene encoding DHODH is located on human chromosome 16 (Fig. 2A). The protein encoded by this gene mainly localizes to the mitochondria and cytoplasm (Fig. 2B). Analysis of protein topology using the PROTTER database revealed the presence of multiple natural missense mutations at amino acids 7, 19, 135, 152, 199, 202, 244, 284, 346, and 392 under physiological conditions (Fig. 2C). The NCBI database was used to examine the baseline expression levels of DHODH in normal human tissues. As shown in Fig. 2D, relatively high mRNA expression was detected in the liver, kidneys, and other organs. To further explore the correlation between DHODH gene expression and tumor diseases, expression data were downloaded from TCGA and GTEx databases. Compared with that in normal tissues, DHODH was significantly upregulated in paired and unpaired tissues of various tumors, including liver cancer, ccRCC, and cholangiocarcinoma (except for MESO) (Fig. 2E, F), aligning with the results from the TIMER database (Supplementary Fig. 1A). The TIMER 2.0 online tool was used to explore the mutation status of DHODH in different tumors and its effect on the prognosis of each tumor. DHODH was found to be mainly present in arm-level deletions and was diploid in multiple tumor types (Supplementary Fig. 1B). In terms of prognosis, DHODH was significantly associated with better outcomes in patients with mesothelioma, renal clear cell carcinoma (Supplementary Fig. 1C), and renal papillary cell carcinoma; however, no significant association was found for the prognosis of patients with liver cancer (Fig. 2G, Supplementary Table 1). Altogether, DHODH was found to be highly expressed in renal cancers (KIRC and KIRP) and was significantly associated with a better prognosis in patients with ccRCC.

### DHODH is highly expressed in renal clear cell carcinoma and is significantly associated with a better prognosis

**Figure 3:**
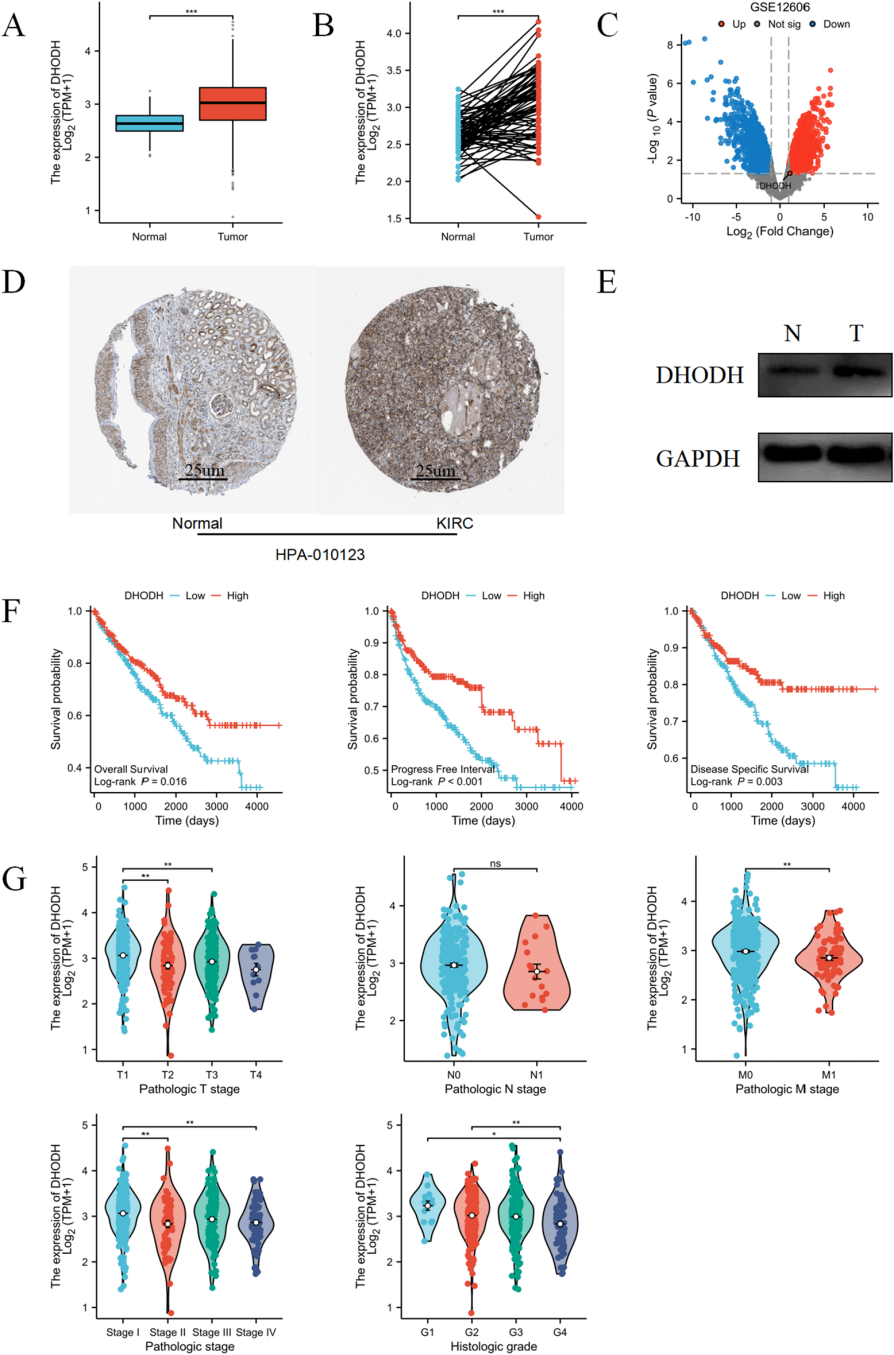
DHODH is highly expressed in ccRCC and indicates a better patient prognosis. (**A**) Differential expression of DHODH in paired samples of ccRCC in TCGA database. (**B**) Differential expression of DHODH in non-paired samples of ccRCC in TCGA database. (**C**) Differentially expressed genes from the ccRCC dataset, GSE12606, in the GEO database. (**D**) Protein expression differences in ccRCC tissues based on the immunohistochemical results obtained from the HPA public database (antibody number HPA-010123). (**E**) Western blot verification of the expression differences in DHODH at the protein level in ccRCC tissues. (**F**) Prognostic analysis of SH3PXD2B expression in patients with ccRCC, specifically OS, PFI, and DSS, using TCGA data. (**G**) Relationship between DHODH expression and T staging, N staging, M staging, pathologic stage, and histologic grade in patients with ccRCC.

Based on the pan-cancer analysis results of DHODH, we analyzed TCGA-KIRC expression profile data in TCGA database. DHODH was significantly upregulated in both paired and unpaired samples of TCGA-KIRC (Fig. 3A, B). In addition, DHODH was highly expressed in the GSE12606 dataset (Fig. 3C). To validate these findings, western blotting was performed using renal clear cell carcinoma tissues and paired normal kidney tissues. DHODH was significantly upregulated in ccRCC tissues (Fig. 3E). The immunohistochemistry results from the HPA database also revealed high expression of DHODH in ccRCC tissues (Fig. 3D). To estimate the role of DHODH expression in predicting the prognosis and progression of ccRCC in patients, we used the R package "stats" to analyze TCGA-KIRC expression profiles and their associated clinical data. High DHODH expression was significantly associated with a better prognosis in patients with ccRCC (Fig. 3F, G), consistent with the above results.

Subgroup survival analysis revealed a trend toward an adverse prognosis associated with increased TNM staging and histological grading in relation to elevated DHODH expression. However, these differences were not statistically significant (Supplementary Fig. 2).

### Correlation between immune cell infiltration and DHODH expression

**Figure 4:**
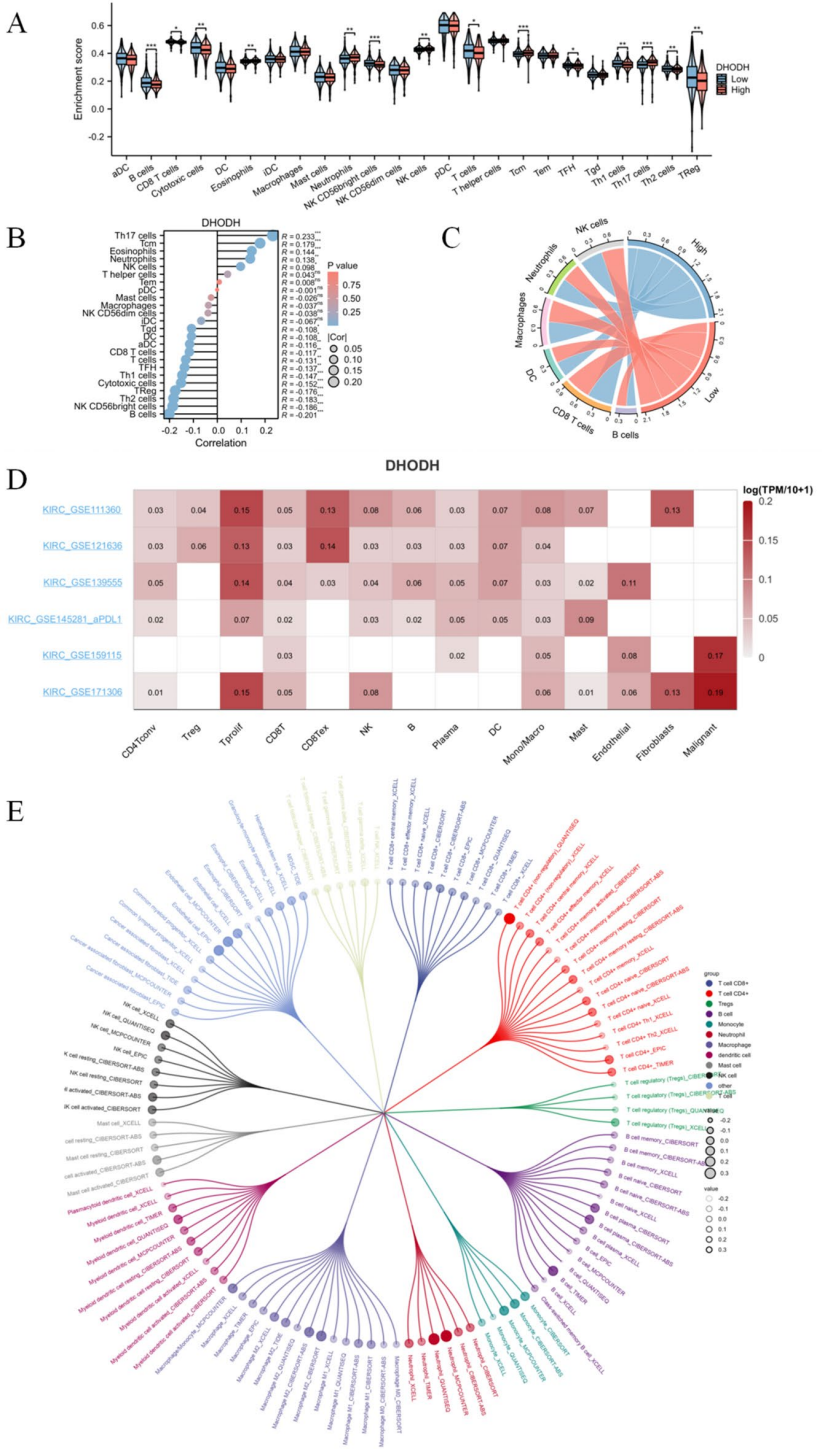
Correlation analysis between immune cell infiltration and DHODH in ccRCC. (**A**) Differences in the infiltration scores of the following 24 immune cell types, activated DC cells (aDC), B cells, CD8 T cells, cytotoxic cells, DC cells, eosinophils, immature DC cells (iDC), macrophages, mast cells, neutrophils, NK CD56bright cells, NK CD56dim cells, NK cells, plasmacytoid DC cells (pDC), T cells, T helper cells, T central memory (Tcm), T effector memory cells (Tem), T follicular helper cells (Tfh), T gamma delta cells (Tgd), Th1 cells, Th17 cells, Th2 cells, and Treg cells between high and low DHODH expression groups. (**B**) Lollipop plots showing the correlation between infiltration scores of 24 immune cell types and DHODH gene expression in patients with ccRCC from TCGA database. (**C**) Chord diagram displaying the correlation between the expression of DHODH and that of six common immune cell types. (**D**) DHODH expression profile in different single cells across multiple GEO datasets. (**E**) Circular dendrogram demonstrating the correlation between DHODH and infiltration of 115 different subtypes of tumor-infiltrating immune cells.

We analyzed the correlation between the infiltration scores for 24 immune cell types and DHODH gene expression in patients with ccRCC in TCGA database. Violin and Laplace plots revealed a significant positive correlation between DHODH expression and the infiltration of immune cells, such as Th17, and a significant negative correlation between DHODH expression and the infiltration of various immune cells, including B cells, NK cells, and tumor-killing cells, such as CD8 + T cells (Fig. 4A, B). A chord diagram comparing the high- and low-expression groups of DHODH revealed similar results (Fig. 4C).

To further examine the cell types expressing DHODH in tumor tissues, we used six datasets from the TISCH database to analyze the single-cell expression of DHODH. Figure 4D shows the relative expression levels of DHODH in 14 cell types, which indicates the widespread expression of DHODH in suppressive immune cells, such as Tregs and malignant cells. By analyzing the effect of DHODH expression on the infiltration of 115 different subtypes of tumor immune cells using the TIMER 2.0 database, a negative correlation was noted between DHODH expression and most immune cell infiltrations (Fig. 4E).

Based on the seven steps in antitumor immune response from the TIP database, we analyzed the differences in the antitumor immune response between samples with high and low DHODH expression. Compared to samples with low DHODH expression (Supplementary Fig. 3B), those with high DHODH expression (Supplementary Fig. 3C) had lower scores for the release of cancer antigen (step 1), cancer antigen presentation (step 2), priming and activation (step 3), trafficking of immune cells to tumors (step 4), T cell infiltration (step 5), and recognition of cancer cells by T cells (step 6) (Supplementary Fig. 3A–C). This result further suggests an immunosuppressive role of DHODH in ccRCC.

We next proceeded to explore the relationship between DHODH and the immune checkpoints. As shown in Supplementary Fig. 3D, DHODH had a significant positive correlation with CD274, CD86, CTLA4, LAG3, CD160, CD209, VEGF-A, and CD274.

### Correlation analysis between DHODH expression and drug sensitivity

**Figure 5:**
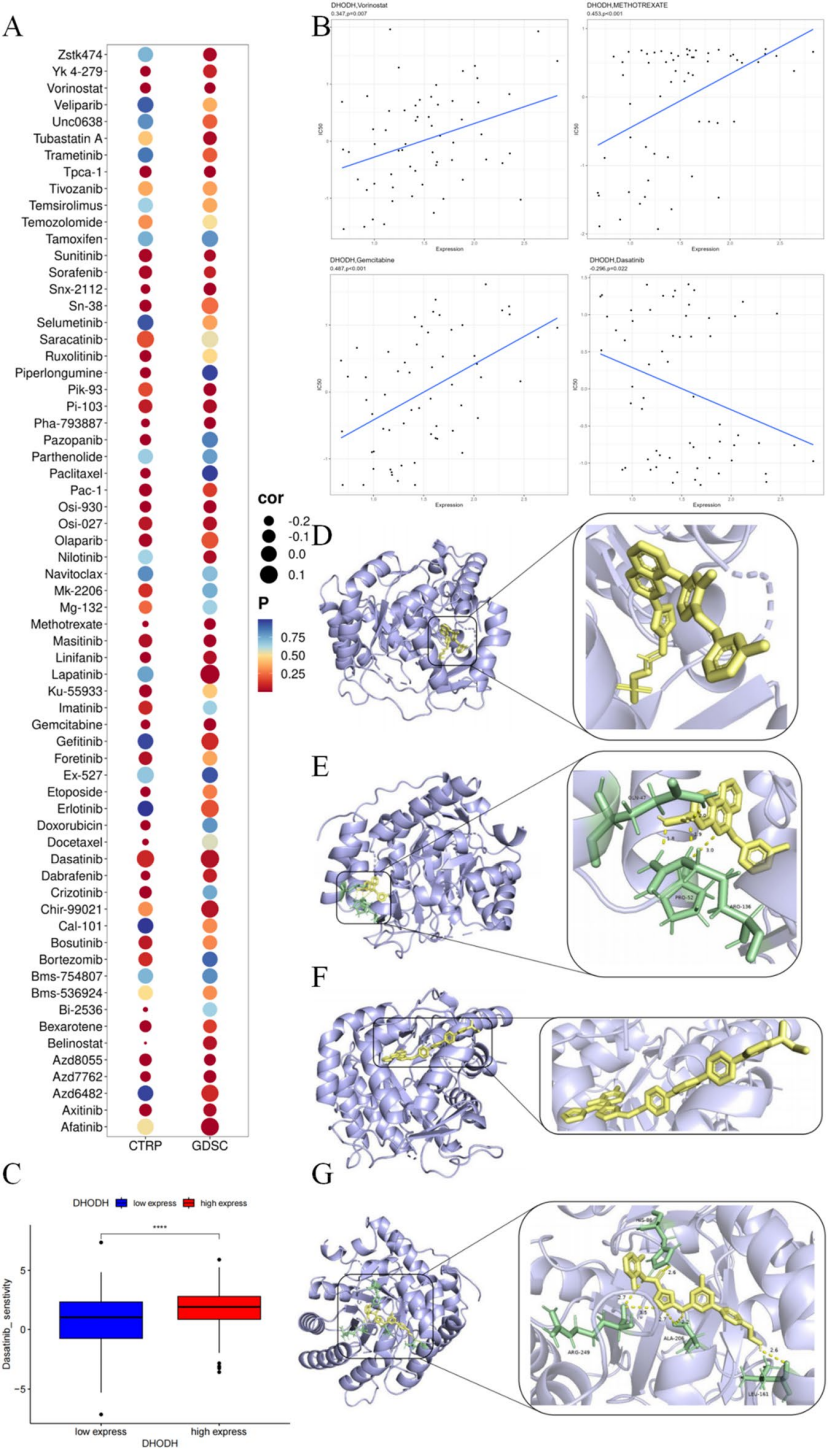
Analysis of drug sensitivity and molecular docking of DHODH. (**A**) Correlation between DHODH expression and sensitivity to various drugs in CTRP and GDSC databases. (**B**) Four drugs significantly associated with DHODH expression in the CellMiner database. (**C**) Box plots demonstrating the sensitivity of dasatinib in samples with high and low DHODH expression. (**D-G**) Molecular docking of DHODH with lapatinib, silmitasertib, itraconazole, and dasatinib, respectively.

By using data from two drug sensitivity databases, GDSC and CTRP retrieved from the GSCALite online database, we determined the correlation between DHODH gene expression and drug sensitivity and applied the high expression and related functions of DHODH genes in ccRCC as potential clinical treatments. Notably, DHODH expression was significantly negatively correlated with sensitivity to multiple small-molecule drugs (Fig. 5A, Supplementary Table 2). In addition, analysis of RNA expression and drug data of NCI-60 cell lines from the CellMiner database revealed that DHODH was significantly positively correlated with the IC50 of vorinostat, methotrexate, and gemcitabine, and significantly negatively correlated with the IC50 of dasatinib (Fig. 5B). The boxplot also highlighted the higher sensitivity of samples with high DHODH expression to dasatinib (Fig. 5C).

Computer molecular docking simulations were performed for drugs that positively correlated with the DHODH protein. Figure 5D–G shows the four drugs with the highest binding affinity: lapatinib (− 11.36 kcal/mol) (Fig. 5D), silmitasertib (− 10.6 kcal/mol) (Fig. 5E), itraconazole (− 10.27 kcal/mol) (Fig. 5F), and dasatinib (− 10.3 kcal/mol) (Fig. 5G).

### Correlation analysis and functional enrichment analysis of DHODH in renal clear cell carcinoma

**Figure 6:**
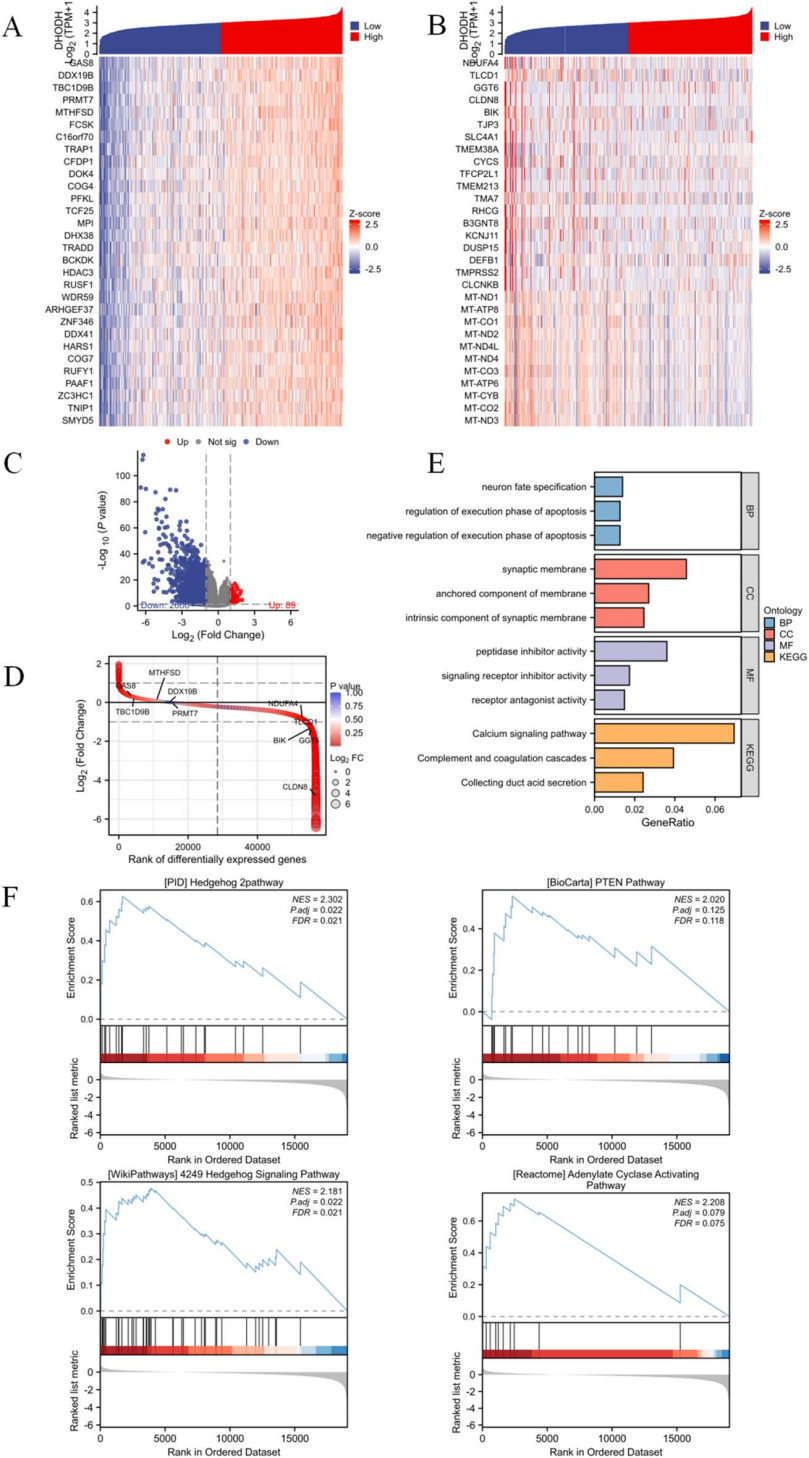
Correlation analysis and functional enrichment analysis of DHODH in ccRCC. (**A**) Top 30 coding genes that positively correlated with DHODH expression at the mRNA level in TCGA database according to Pearson correlation coefficient. (**B**) Top 30 coding genes that negatively correlated with DHODH expression at the mRNA level in TCGA database according to Pearson correlation coefficient. (**C**) Gene volcano plot of differential expression between high and low DHODH expression groups in TCGA-KIRC samples. (**D**) Ranking map of genes differentially expressed between high and low DHODH expression groups in TCGA-KIRC samples (ranked by lgFC). (**E**) KEGG enrichment analysis of the differentially expressed genes associated with DHODH. (**F**) Gene set enrichment genes of DEGs associated with DHODH.

To further explore the potential functions of DHODH in ccRCC, a correlation analysis was conducted between DHODH and all other mRNAs in ccRCC using TCGA data. The top 30 positively and negatively correlated genes are presented in Fig. 6A, B. Volcano and gene ranking plots were generated to illustrate the DEGs between the high- and low-expression groups of DHODH in TCGA-KIRC samples (Fig. 6C, D).

GO and KEGG enrichment analyses were performed for the DEGs identified from the single-gene differential analysis. Based on GO functional enrichment analysis, DHODH was primarily associated with pathways such as the negative regulation of the execution phase of apoptosis. The KEGG analysis results revealed a close correlation between DHODH and pathways such as the calcium signaling pathway (Fig. 6E, Supplementary Table 3).

GSEA was performed using the DEGs associated with DHODH. Hedgehog signaling, adenylate cyclase activation, and PTEN pathways were found to be significantly enriched. These findings suggest a correlation between the high expression of DHODH and multiple carcinogenic pathways in ccRCC (Fig. 6F).

### Identification of a ceRNA network

**Figure 7:**
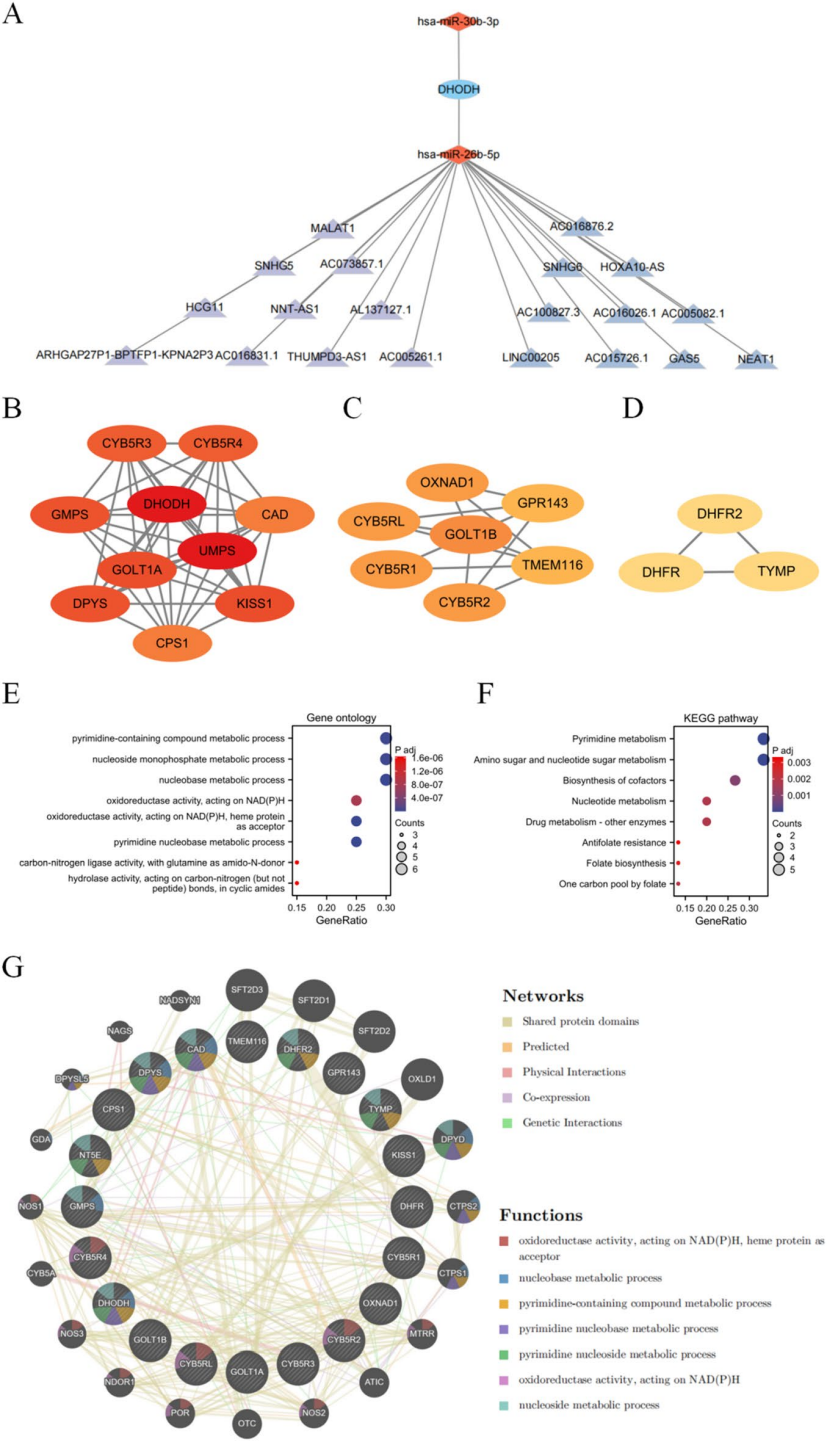
The regulatory relationship of DHODH. (**A**) lncRNA-miRNA-mRNA network; the construction of a ceRNA network. (**B-D**) Highly connected target mRNAs were identified using MCODE. (**E–F**) GO and KEGG analyses of proteins interacting with DHODH. (**G**) DHODH co-expression network and function analysis based on the GeneMANIA database.

We used mirTarBase to predict 115 miRNAs that could bind to the DHODH gene (Supplementary Table 4). Differential analysis of GSE189331 using the Limma [3.5.2] package revealed significant differences in miRNA expression among the 115 candidates. In particular, hsa-miR-30b-3p, hsa-miR-26b-5p, and hsa-miR-4455 had significant differential expression; hsa-miR-30b-3p and hsa-miR-26b-5p were significantly downregulated whereas hsa-miR-4455 was significantly upregulated in the sunitinib-resistant group (Supplementary Fig. 4A). GSCALite analysis revealed a negative correlation between DHODH and sunitinib sensitivity. Next, we predicted the potential lncRNAs that could interact with hsa-miR-30b-3p and hsa-miR-26b-5p using the StarBase database, which led to the identification of 20 lncRNAs (Supplementary Table 5). Among them, AL137127.1, THUMPD3-AS1, NNT-AS1, HCG11, SNHG5, AC016831.1, MALAT1, AC073857.1, ARHGAP27P1-BPTFP1-KPNA2P3, and AC005261.1 were significantly associated with a poor prognosis in patients with ccRCC (Supplementary Fig. 4B). Therefore, we preliminarily established a DHODH-miRNA-lncRNA ceRNA network (Fig. 7A) that may have significant implications for ccRCC progression.

Using the STRING database, we constructed a medium confidence (> 0.4) protein interaction network for DHODH, which comprised 21 nodes and 130 interaction pairs. Three densely connected gene modules were identified using the MCODE plugin in Cytoscape (Fig. 7B–D). GO analysis revealed a significant enrichment of these genes in processes, such as nucleobase metabolism (Fig. 7E), whereas KEGG pathway analysis revealed their involvement in pathways, such as amino sugar and nucleotide sugar metabolism (Fig. 7F), indicating a potential association of DHODH with nucleotide metabolism.

We next analyzed the co-expression network and functional relevance of these genes using the GeneMANIA database. These genes exhibited a complex network of protein–protein interactions, including 51.80% shared protein domains, 29.73% predicted interactions, 11.00% physical interactions, 7.05% co-expression, 11.58% physical interactions, and 0.42% genetic interactions (Fig. 7G).

### DHODH influences the proliferation and migration of ccRCC cells

**Figure 8:**
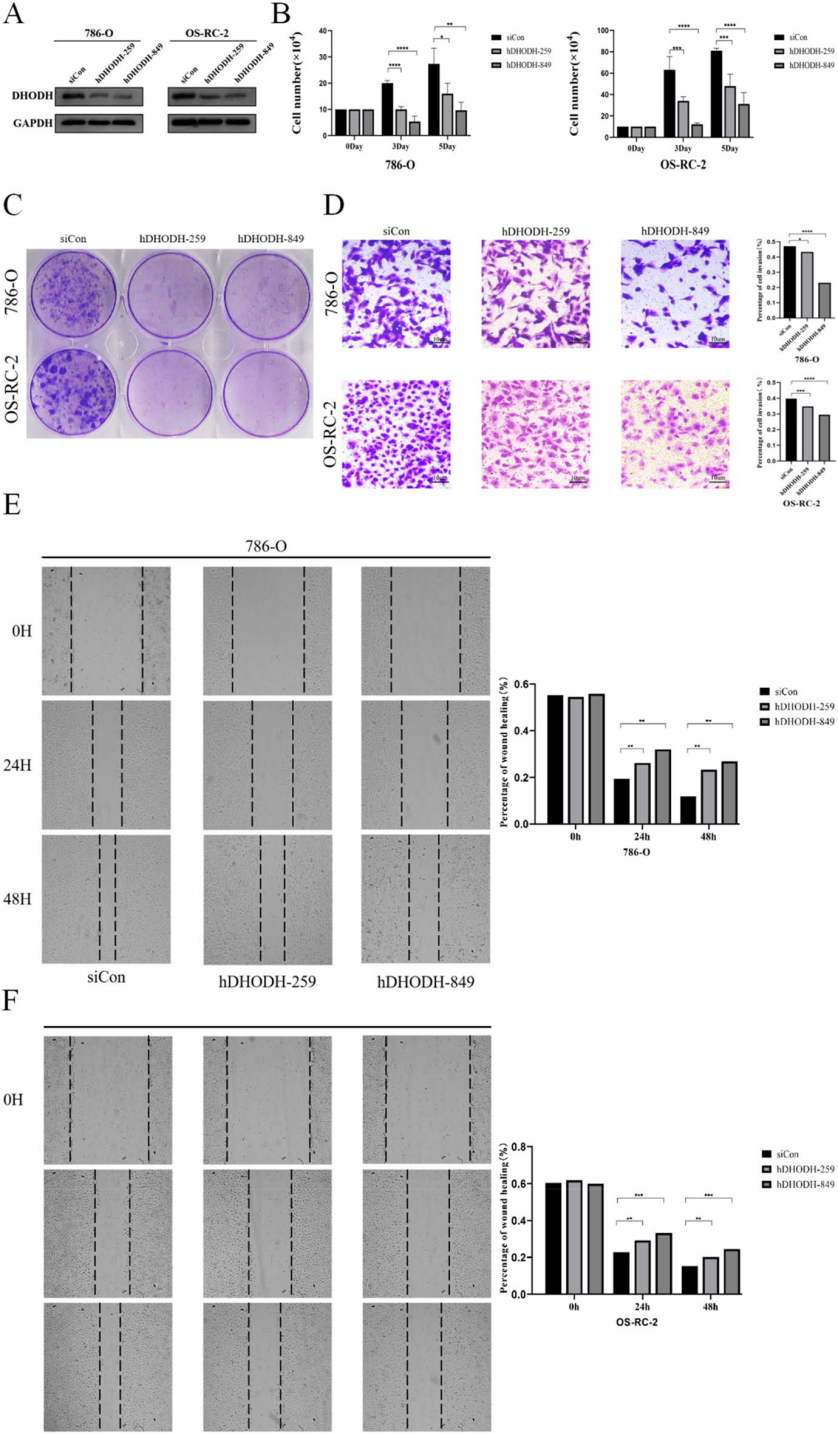
DHODH knockout attenuates the proliferation and migration of 786-O and OS-RC-2. (**A**) The expression of DHODH following knockout based on western blot analysis. (**B**) 786-O and OS-RC-2 cells were transfected with the control or DHODH siRNA and counted on days 0, 3, and 5. (**C**) 786-O and OS-RC-2 cells were transfected with the control or DHODH siRNA, and cell proliferation was detected via colony formation. (**D**) 786-O and OS-RC-2 cells were transfected with the control or DHODH siRNA, and cell invasion ability was analyzed using the transwell assay. (**E–F**) 86-O and OS-RC-2 cells were transfected with the control or DHODH siRNA, and cell migration ability was analyzed using the cell scratch assay. *P < 0.05, **P < 0.01, ***P < 0.001, ****P < 0.0001. The asterisk represents the degree of statistically significant difference (*P) (n = 3).

As cell proliferation and migration are critical phenotypes for tumor progression, we examined the effects of DHODH gene knockdown on ccRCC cells in vitro. Stable, low expression of DHODH was successfully established in 786-O and OS-RC-2 ccRCC cell lines through lentiviral transduction (Fig. 8A). Cell functional assays, including proliferation, migration, and colony formation experiments, were performed using shDHODH and corresponding empty vector harboring control cells.

**Figure 9:**
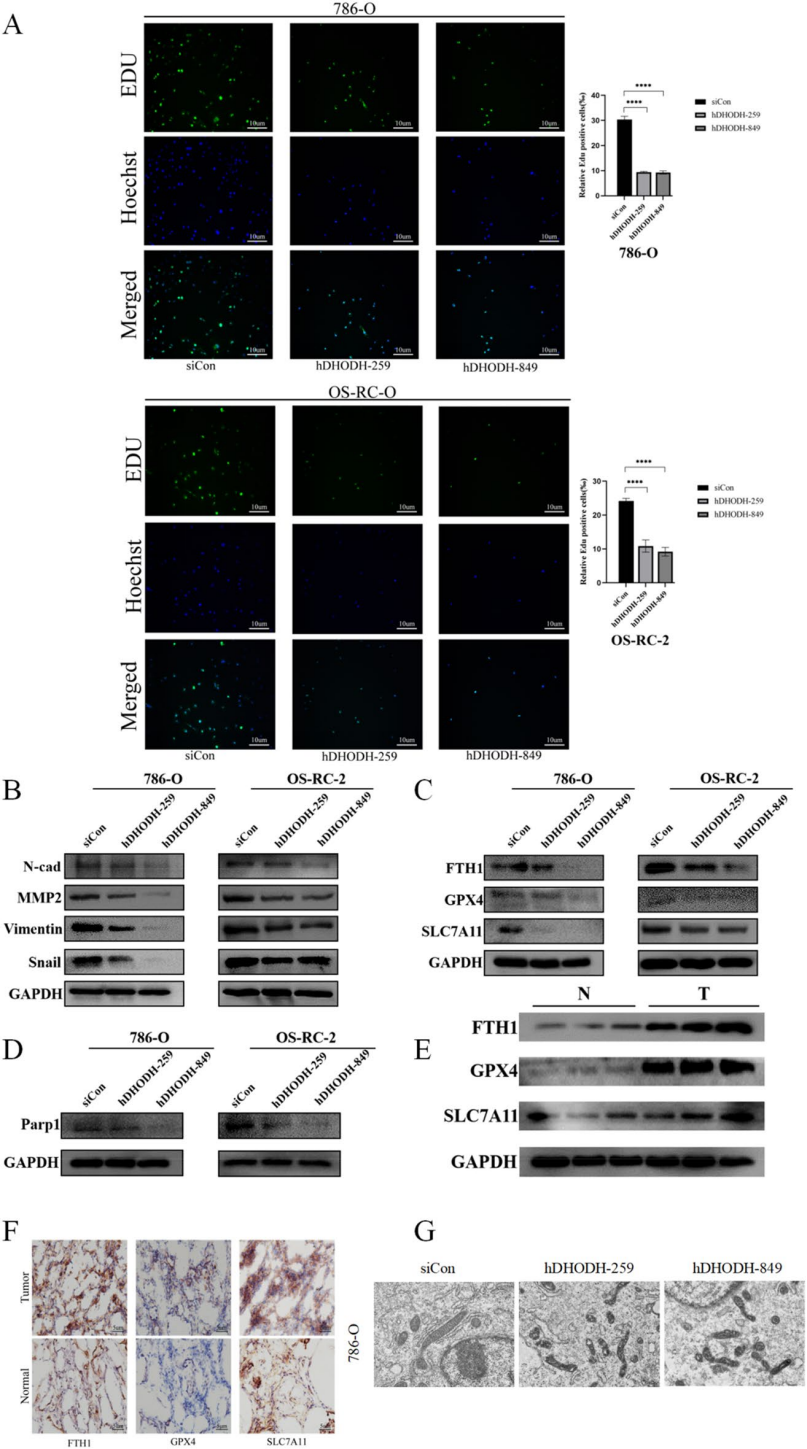
DHODH knockout attenuates the proliferation, migration, and ferroptosis of 786-O and OS-RC-2 cells. (**A**) Knockdown of DHODH in 786-O and OS-RC-2 cells and evaluation of the cell proliferation ability using the EDU assay. (**B**) Western Blotting was performed to determine the expression of EMT-related proteins in 786-O and OS-RC-2 cells transfected with DHODH or the control for 48 h. (**C**) Western blot analysis was performed to determine the expression of ferroptosis-related proteins in 786-O and OS-RC-2 cells after knockdown of DHODH. (**D**) Western blot analysis was performed to determine the expression of the apoptotic protein, Parp-1, after DHODH knockdown. (**E**) Expression of ferroptosis-related protein in ccRCC tissues and normal renal tissues. (**F**) Immunohistochemistry was performed to visualize the expression of ferroptosis-associated protein in normal renal tissue and renal clear cell carcinoma. (**G**) Changes in the mitochondria of renal clear cancer cells after DHODH knock down via transmission electron microscopy. Scale bar represents 1 µm (× 12000). *P < 0.05, **P < 0.01, ***P < 0.001, ****P < 0.0001. The asterisk represents the degree of statistically significant difference (n = 3).

Cell counting, EDU labeling, and colony formation assays revealed that the proliferation and colony formation abilities of both cell types were significantly weakened after DHODH knockdown (Figs. 8B–D and 9A). Similarly, according to the results of the transwell and scratch assays, the migration ability of ccRCC cells was significantly decreased after DHODH knockdown (Fig. 8E, F). Such finding suggests that DHODH promotes the development of ccRCC.

### DHODH promotes epithelial-mesenchymal transition (EMT) and inhibits ferroptosis

EMT is a crucial factor that contributes to tumor invasion and metastasis. In our experiments, DHODH downregulation inhibited the expression of the EMT-related proteins, N-cadherin, vimentin, Snail, and MMP2, in ccRCC cells (Fig. 9B). Furthermore, GO enrichment analysis indicated an association between DHODH and apoptosis regulation, which was supported by the western blot results, in which DHODH siRNA was found to downregulate the expression of the apoptosis-related protein, Parp1 (Fig. 9D). By searching the GeneCard database, we discovered that DHODH is associated with ferroptosis. To explore the mechanism of action of DHODH in the ferroptosis of clear cell RCC cells, we conducted in vitro experiments. Downregulation of DHODH inhibited the expression of FTH1, GPX4, and SLC7A11 in 786-O and OS-RC-2 cells (Fig. 9C). Moreover, western blot analysis revealed a significant increase in the expression of FTH1, GPX4, and SLC7A11 in ccRCC tissues compared to those in normal kidney tissues (Fig. 9E, F). In DHODH knockdown ccRCC cells, typical morphological features of ferroptosis were observed using transmission electron microscopy, including mitochondrial atrophy, shrinkage, and increased membrane density (Fig. 9G). Overall, these findings provide evidence that DHODH may suppress the ferroptosis of ccRCC cells by promoting the negative regulation of the iron death-associated proteins, FTH1, GPX4, and SLC7A11.

## Discussion

According to prior studies, DHODH is associated with the development of various tumors, including breast cancer^16^ and liver cancer^18^. However, the mechanism of action of DHODH on ccRCC has remained unclear. Therefore, to explore the effect of DHODH on the occurrence and development of ccRCC and determine whether it can be used as a potential therapeutic target for ccRCC, we opted to conduct bioinformatics analysis and in vitro experimental verification.

Using various databases, DHODH was identified to be commonly upregulated in various malignant tumors, including ccRCC. Similarly, western blot analysis of paired tissues from patients with ccRCC revealed high DHODH expression in ccRCC tissues. To determine the correlation between DHODH and the prognosis of patients with ccRCC, the clinical characteristics of these patients were retrieved from TCGA database. Notably, we found a significant positive correlation between high expression of DHODH and better prognosis in patients with ccRCC. The high expression of DHODH in ccRCC and its association with better prognosis is expected to garner significant interest in further exploring the mechanism of DHODH in ccRCC.

Immune cells play an extremely important role in the occurrence and development of tumors^38^. In recent years, immunotherapy has become an effective treatment for malignant tumors, particularly advanced ccRCCs^39^. To evaluate the effect of DHODH on immune cells in ccRCC, we used multiple databases to conduct immune infiltration analysis and consistently found a negative correlation between DHODH and the infiltration of tumor-killing cells, such as B cells, NK cells, and CD8 + T cells. Furthermore, DHODH reduced the activity of the seven steps of the antitumor immune response, and its expression was positively correlated with multiple immune checkpoints. These findings indicate that DHODH may inhibit immune cell activity in ccRCC. Therefore, DHODH is a potential biomarker for predicting the clinical benefits of immunotherapy.

Despite the widespread T cell infiltration observed in RCC, novel therapeutic approaches, such as targeted therapy and immunotherapy, have improved the survival rates of patients with RCC. However, the efficacy of these therapies remains limited, with persistent immune suppression and resistance observed in the tumor microenvironment (TME). Therefore, it is imperative to identify new therapeutic targets to enhance the efficacy of immunotherapeutic agents for RCC^40–42^. Notably, targeting DHODH is a potential strategy for anti-ccRCC immunotherapy. A negative correlation was found between DHODH expression and sensitivity to various small-molecule drugs. Sensitivity to dasatinib was significantly higher in the high DHODH-expression group. Dasatinib is an orally administered potent adenosine triphosphate competitive inhibitor that inhibits multiple tyrosine kinases and has been used in various solid tumors^43^ to inhibit tumor cell proliferation and invasion^44^. Lapatinib is an orally administered, reversible epidermal growth factor receptor (EGFR)/human epidermal growth factor receptor 2 (HER-2) tyrosine kinase inhibitor. Clinical studies have revealed that lapatinib had promising results regarding RCC treatment^45,46^. The Ser/Thr protein kinase, CK2, is involved in important pathways related to tumor progression, such as Hedgehog and Wnt^47^. Silmitasertib (CX-4945) is an inhibitor of CK2, and its efficacy has been confirmed in many malignant tumors as it blocks the cell cycle of various tumor cells and inhibits tumor cell proliferation, migration, and invasion^48^. Recent studies have also found that itraconazole exerts inhibitory effects on various solid tumors and hematologic malignancies by inhibiting angiogenesis and various oncogenic signaling pathways, such as the Hedgehog signaling pathway^49^. Our molecular docking results revealed that the four aforementioned drugs bound strongly to the DHODH protein, suggesting the potential treatment of ccRCC by targeting DHODH. We ascertained the clinical significance of DHODH in sensitizing ccRCC to drug therapies and propose DHODH as a promising molecular target for future immunotherapeutic or targeted treatments.

To comprehensively analyze the function of DHODH in ccRCC, we conducted functional enrichment analysis of the DEGs identified from single-gene differential analysis using DHODH. Our findings suggest that DHODH may be involved in the negative regulation of tumor cell apoptosis. GSEA also revealed significant enrichment in pathways, such as the Hedgehog signaling and PTEN pathways. Dysregulation of the Hedgehog signaling pathway has been associated with various tumors, including basal cell carcinoma, medulloblastoma, pancreatic cancer, breast cancer, colon cancer, and small-cell lung cancer. Abnormal activation of the Hedgehog signaling pathway has been detected in multiple types of tumors, leading to malignant transformation^50^. *PTEN* is a tumor suppressor gene, and mutations in *PTEN* have been found in various tumors, including endometrial, skin, and prostate tumors^51^. Accordingly, DHODH may be involved in the occurrence and development of ccRCC.

To investigate the potential regulatory mechanisms of DHODH in ccRCC, we constructed a PPI network and a DHODH-hsa-miR-26b-5p-lncRNA ceRNA network. In the PPI network, DHODH was found to interact with UMPS and CAD to form enzyme complexes, facilitating pyrimidine biosynthesis in cells, thereby promoting DNA synthesis to regulate tumor cell proliferation and inhibit ferroptosis^52^. In gastric cancer, CAD and DHODH can accelerate glycolysis, thereby enhancing chemotherapy resistance. However, inhibiting the pyrimidine biosynthesis pathway mediated by CAD and DHODH through drug intervention can render tumor cells sensitive to chemotherapy^53^. Inhibiting DHODH can also deplete the cellular pyrimidine nucleotide supply to suppress the survival, proliferation, and tumorigenesis of glioblastoma stem cells. Therefore, targeting the inhibition of CAD, UMPS, and DHODH is a promising approach for ccRCC treatment^54,55^.

In the ceRNA network, we predicted that hsa-miR-26b-5p and hsa-miR-30b-3p may be the target molecules of DHODH; however, no direct evidence of their interaction exists. Studies have shown that miR-30b-5p inhibits lung cancer cell proliferation, migration, and invasion by targeting LRP8 and enhancing lung cancer cell sensitivity to cisplatin^56^. miR-30b-5p can also inhibit EMT by targeting Rap1b, thereby suppressing colorectal cancer metastasis^57^. Meanwhile, hsa-miR-26b-5p directly interacts with PDE4B and CDK8, thereby downregulating STAT3 phosphorylation and nuclear translocation, and promoting gastric cancer cell proliferation^58^. hsa-miR-26b-5p downregulates MYCBP expression, ultimately inhibiting cell proliferation and EMT in triple-negative breast cancer^59^. These findings suggest that hsa-miR-26b-5p and miR-30b-5p may mediate the malignant phenotype of ccRCC by targeting downstream molecules. We predicted 10 potential target lncRNAs of hsa-miR-26b-5p that may affect the prognosis of patients with ccRCC. Overall, DHODH may promote ccRCC progression by targeting downstream lncRNAs and mRNAs through hsa-miR-26b-5p.

By performing cell experiments to further verify whether DHODH promotes RCC, we found that DHODH knockdown significantly reduced the proliferation, migration, and invasion of ccRCC cells. Western blot analysis also revealed a decrease in the expression of apoptosis-related proteins after DHODH knockdown, confirming that DHODH inhibits the apoptosis of ccRCC cells. DHODH promoted EMT and inhibited the ferroptosis of ccRCC cells, thereby promoting ccRCC progression. However, these findings contradicted those of our survival analyses. In our subgroup survival analysis, we observed a gradual trend toward poor prognosis associated with increased DHODH expression due to advances in the TNM staging and histological grading. Although this trend was not statistically significant, which might be due to the limited sample size of higher TNM stages and histological grades, it may mirror the effect of TGF-β on tumorigenesis, akin to the multistep tumorigenesis model proposed by Vogelstein et al.^60,61^. According to this model, tumorigenesis unfolds through successive genetic alterations over time, enabling molecules to exert both anti- and pro-tumorigenic effects at different stages of tumor development. Prior studies have indicated a dual role of TGF-β in breast cancer, initially exerting tumor-suppressive effects in early stages and transitioning to pro-tumorigenic effects in later stages^62^. As our survival analysis relied on transcriptomic data from TCGA database, the discordance between mRNA and protein expression patterns and biological mechanisms was elucidated^63^. Consequently, potential disparities may exist between the regulatory effects of DHODH expression at the RNA level, and its effects on ccRCC development and patient survival at the protein level. Considering that a multitude of factors including age, ethnicity, and environmental factors influences patient survival, and the potential bias introduced by a survival analysis that solely addressed the correlation between DHODH expression and patient outcomes without accounting for multifactorial influences, additional studies are warranted. Moreover, by acknowledging the inherent limitations of relying solely on publicly available online databases for analysis and validation, further explorative, multicenter, prospective studies should be conducted^64^. In future studies, we aim to delve deeper into the specific mechanisms underlying the effect of DHODH on different stages of ccRCC development and validate its prognostic significance in patients with ccRCC through additional experimental designs and prospective studies.

## Conclusion

This study provides multidimensional evidence based on multiple databases, analytical methods, and in vitro experiments. We found that DHODH plays a crucial regulatory role in immune cell infiltration, drug sensitivity, proliferation, migration, invasion, EMT, and ferroptosis of ccRCC cells. Overall, our findings suggest that DHODH can promote ccRCC progression and may be an effective target for the clinical treatment of ccRCC.

### Supplementary Information

Supplementary Figures.

Supplementary Table 1.

Supplementary Table 2.

Supplementary Table 3.

Supplementary Table 4.

Supplementary Table 5.

## Data Availability

The data provided in this study can be obtained from the article materials and supplementary information files.
