# Supplementary material for: Bioinformatics analysis and experimental verification of the cancer-promoting effect of DHODH in clear cell renal cell carcinoma
Source: Sci Rep. 2024 May 25;14:11985. doi: 10.1038/s41598-024-62738-0 (PMC11127953; doi:10.1038/s41598-024-62738-0)
Supplement: Supplementary file 1 — Supplementary Figures. [file 41598_2024_62738_MOESM1_ESM.docx]

**Bioinformatics analysis and experimental verification of the cancer-promoting effect of DHODH in clear cell renal cell carcinoma**

**Songsong Wang^1,2#^, Yan Li^3,4#^, Yilong Lin^2,5#^, Junting Li^6^, Lang Guo^3,4^, Haoyu Wang^3,4^, Xinyuan Lin^6^, Ziming Liu^1✉^, Bingqi Zhang^3,4✉^,** **Zhengming Liao^3,4^****^✉^, Zhongmin Zhang^3,4✉^**


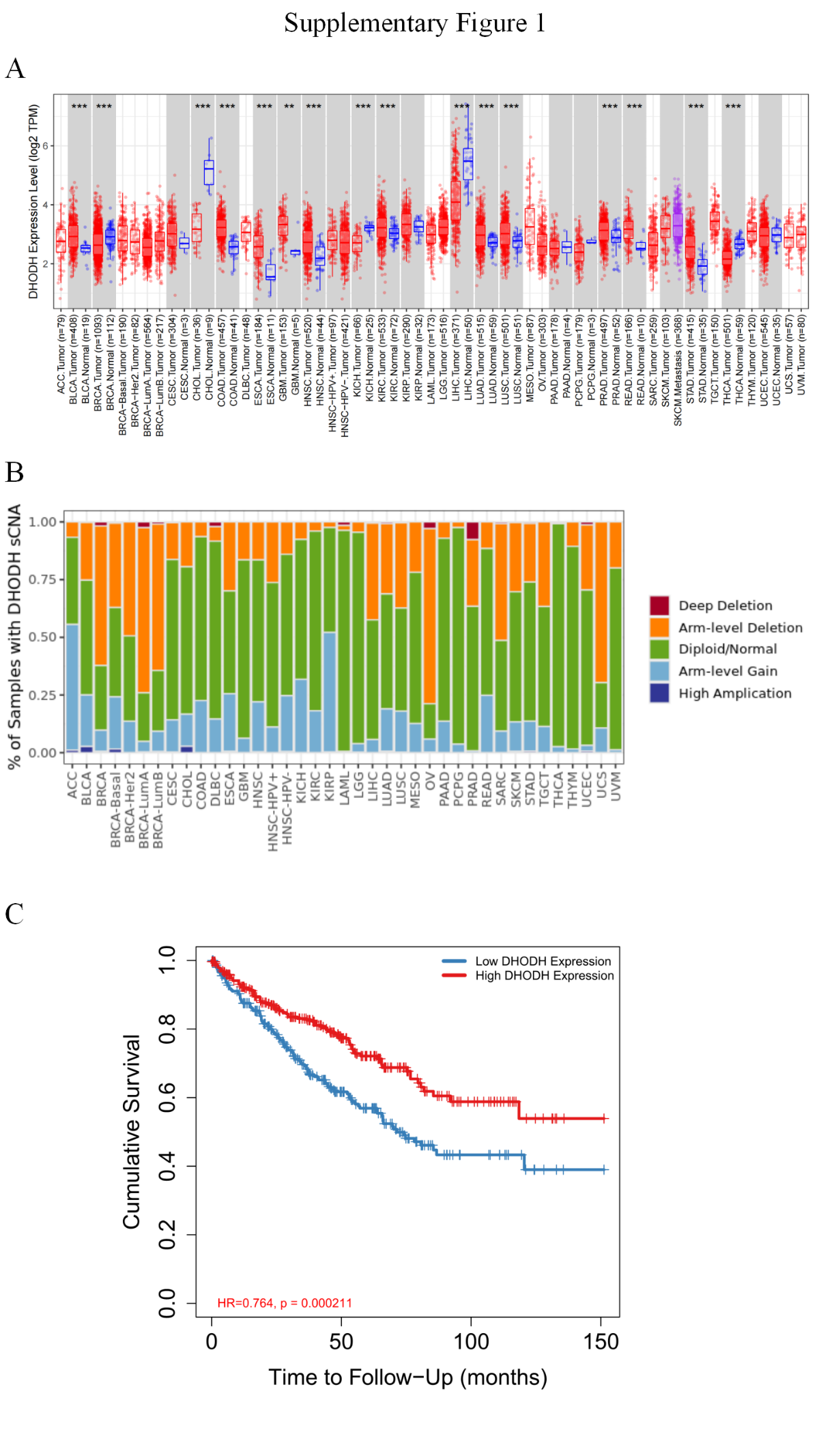


**Supplementary Figure 1.** (**A**) mRNA expression levels of DHODH were analyzed in different cancer types from TCGA data in TIMER2.0. *p<0.055, **p<0.01, ***p<0.001. (**B**) The sCNA of DHODH were analyzed in different cancer types from TCGA data in TIMER2.0. (**C**) Survival of DHODH in ccRCC from TCGA data in TIMER2.0.


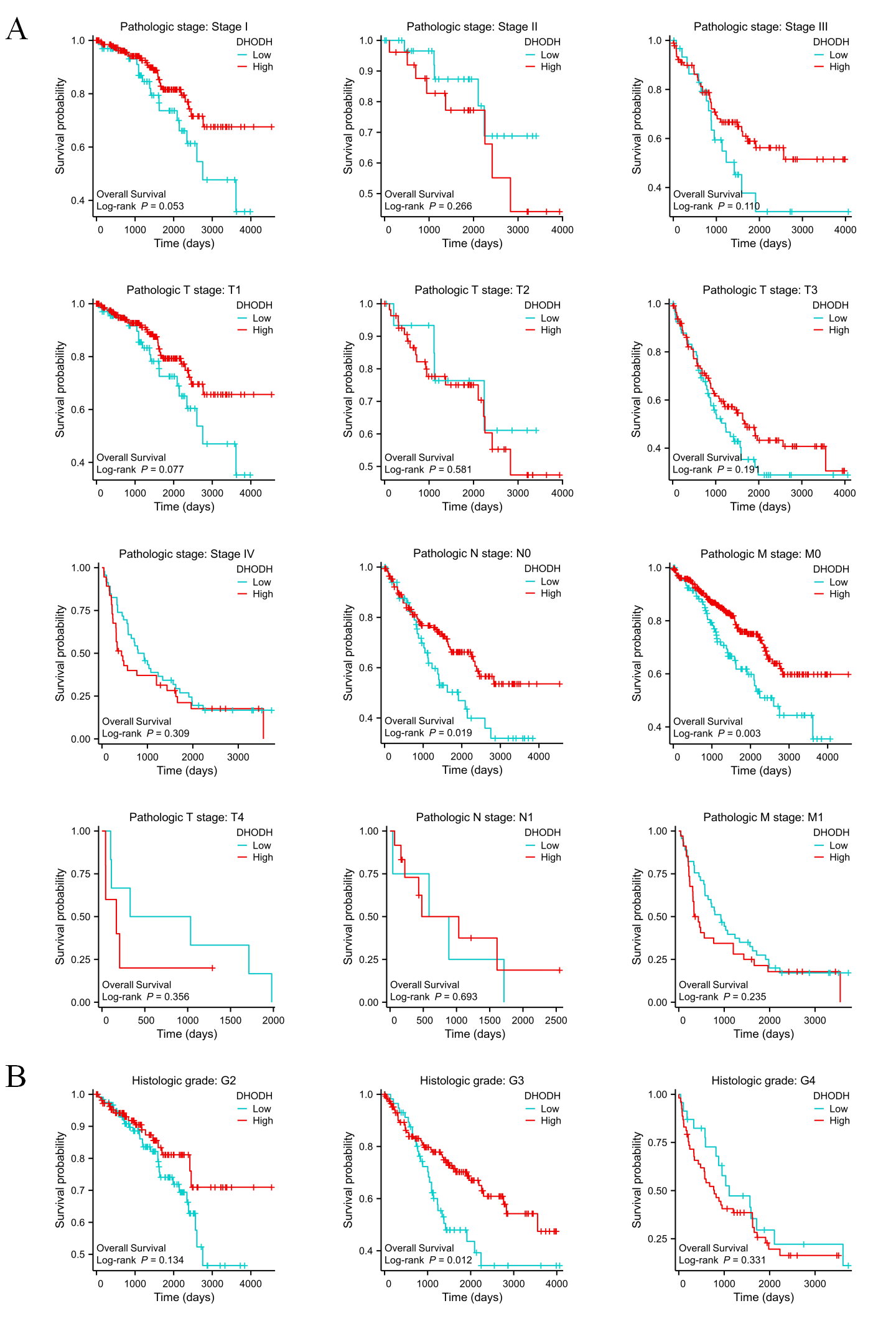


**Supplementary Figure 2.** (**A**) Subgroup survival analysis by TNM stage. (**B**) Subgroup survival analysis by histologic grade.


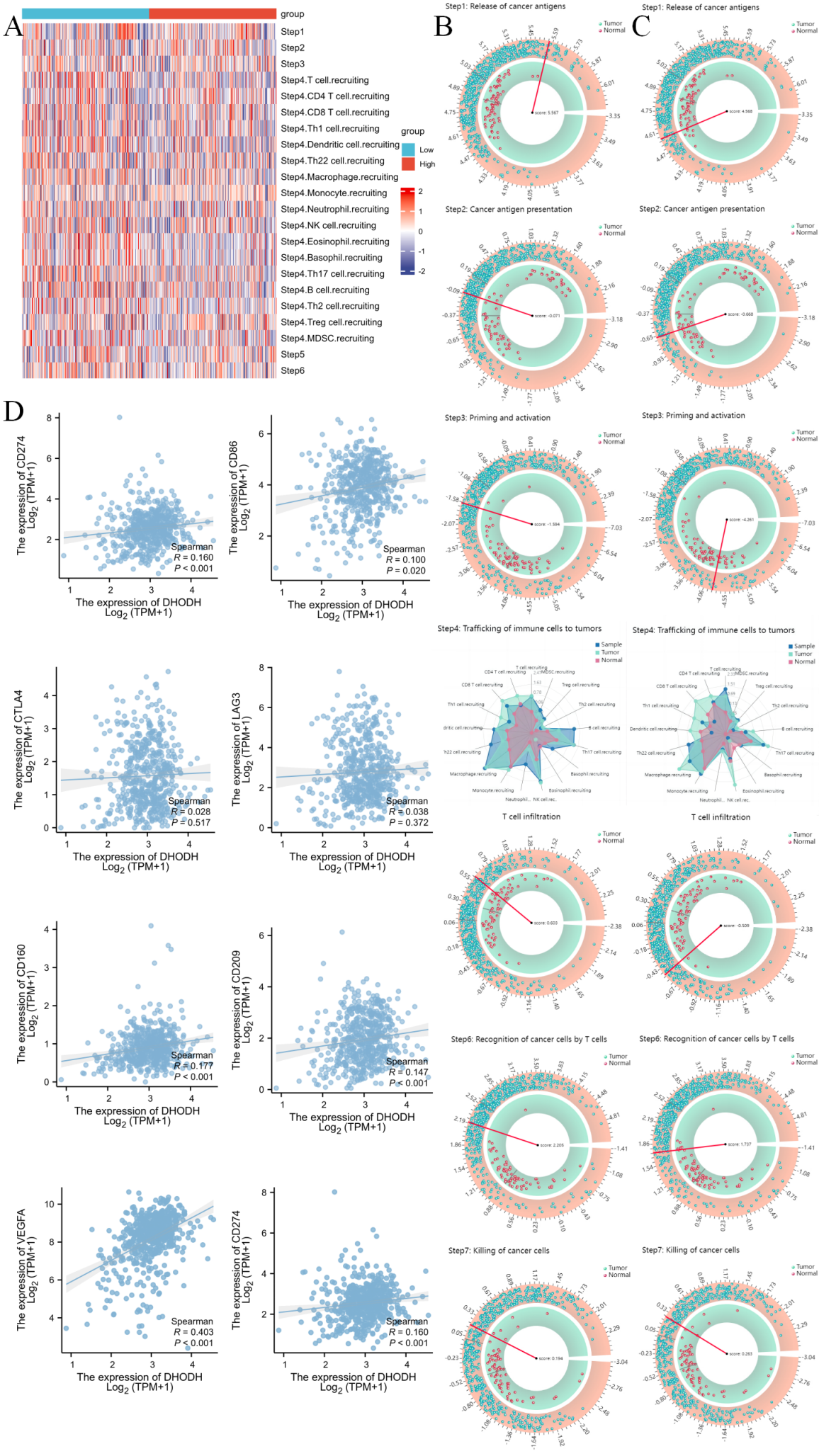


**Supplementary Figure 3.** Correlation analysis between immune response, immune checkpoints and DHODH. (**A**) Heatmap showing the correlation between DHODH expression and activity scores of the cancer immune cycle in TCGA samples. (**B-C**) Activity scores of the cancer immune cycle in samples with low and high DHODH expression. (**D**) Scatter plot displaying the correlation between immune checkpoint expression and DHODH expression.


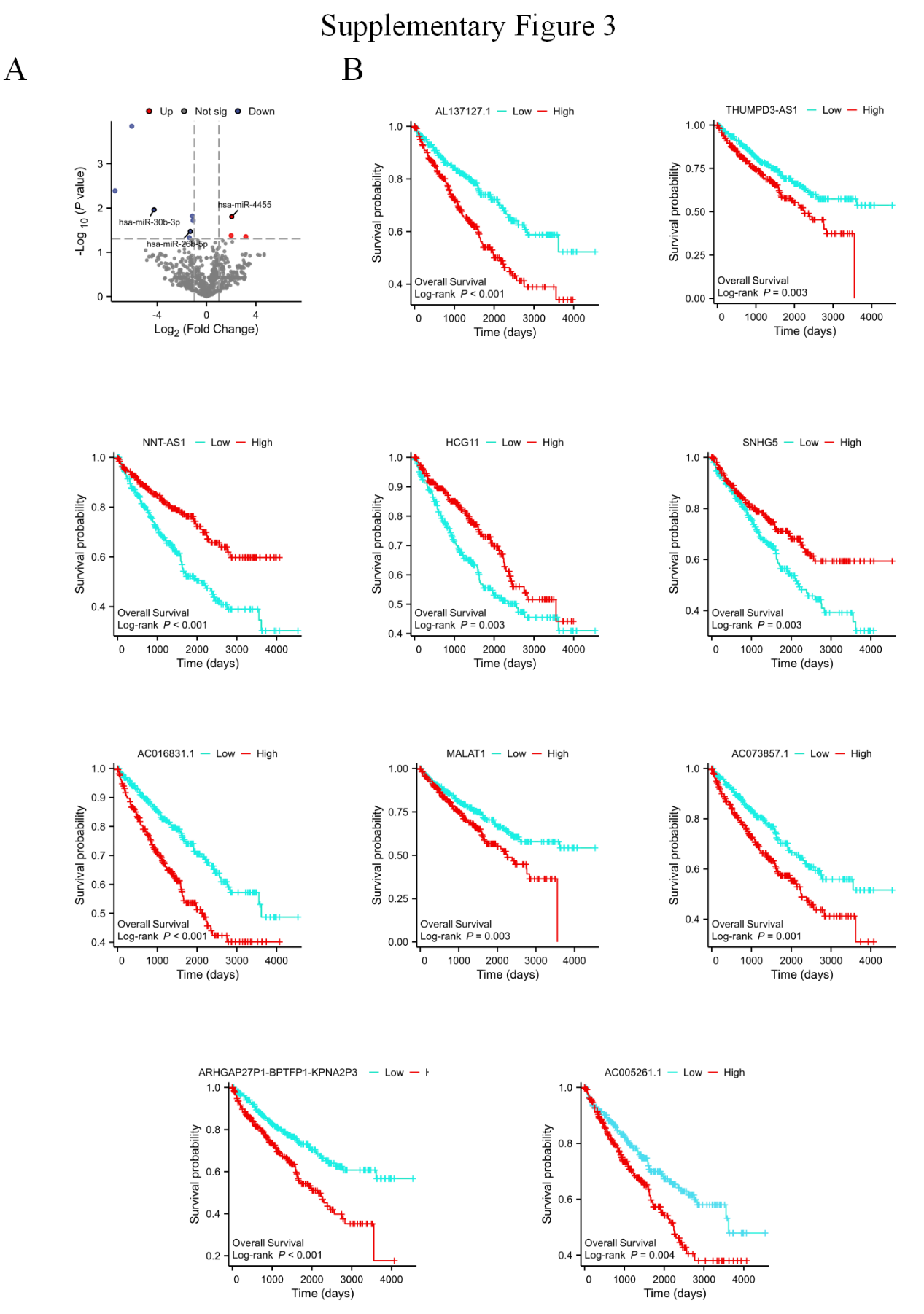


**Supplementary Figure 4.** (A). Differential analysis of GSE189331. (B) Survival of lncRNAs in ccRCC from TCGA data.
